# Social and emotional performance of deaf and hard-of-hearing students in inclusive schools: a mixed-methods analysis of teachers' experiences in Saudi Arabia

**DOI:** 10.3389/fpsyg.2026.1828336

**Published:** 2026-05-29

**Authors:** Nourah Ibrahim Albash

**Affiliations:** Special Education Department, College of Education, King Faisal University, Al-Ahsa, Saudi Arabia

**Keywords:** emotional learning, hearing impairment, inclusive education, Saudi Arabia, social learning

## Abstract

Social and emotional performance is a fundamental component in promoting the psychological and social adjustment of deaf and hard-of-hearing (DHH) students, as it contributes to the development of communication skills, emotional regulation, and the formation of positive interpersonal relationships. The present study aimed to examine teachers' perceptions of the social and emotional performance of deaf and hard-of-hearing students in inclusive schools. The study employed a mixed-methods approach using a sequential explanatory design. Quantitative data were collected through the administration of a Social and Emotional Performance Scale to 102 teachers of deaf and hard-of-hearing students in Saudi Arabia. In addition, semi-structured interviews were conducted with 15 teachers who were purposively selected from among the survey participants to obtain in-depth insights. The quantitative findings indicated that the level of social and emotional performance among deaf and hard-of-hearing students, from the teachers' perspectives, was moderate. The results also showed no statistically significant differences in teachers' perceptions attributable to the variables of academic qualification, years of teaching experience, educational stage, or teaching category. The qualitative findings revealed three main factors influencing the social and emotional performance of DHH students: family, peers, and teachers; communication methods used; and the educational environment and assistive resources. The findings also identified a set of strategies proposed by teachers to enhance students' social and emotional performance, including promoting emotional expression and emotional regulation in challenging situations, addressing frustration and academic failure, participation in programs and activities that support psychological adjustment, using sign language and assistive technologies, encouraging friendships and addressing bullying and social exclusion, developing cooperation and teamwork skills, and implementing school initiatives that promote community acceptance. Overall, the findings highlight the importance of strengthening social and emotional support strategies and developing inclusive educational environments that foster the social integration and school adjustment of deaf and hard-of-hearing students.

## 1 Introduction

According to estimates by the World Health Organization, approximately 34 million children worldwide experience deafness or hearing loss exceeding 40 decibels (World Health Organization, 2019). Hearing loss affects not only auditory functioning but also multiple aspects of an individual's life, particularly communication and social interaction. As a result, individuals who are deaf or hard of hearing (DHH) often experience social isolation and loneliness due to difficulties in interacting with others, which may negatively affect their psychological and social wellbeing compared with their hearing peers (Bennett et al., 2022; Heffernan et al., 2016; Timmer et al., 2024). Research further indicates that hearing loss may affect individuals' ability to accurately perceive and interpret emotional cues, which may consequently influence their social and emotional functioning (Picou et al., 2018).

Given these challenges, the educational context plays a critical role in supporting the social and emotional development of DHH students. When these needs are not adequately addressed, their effects may manifest through behaviors observed by teachers in the classroom, highlighting the importance of integrating social and emotional learning within educational practices, particularly in inclusive educational settings (Herzig and Leannah, 2022). Most DHH students receive their education in mainstream schools through inclusive education programs, where they receive additional educational support from teachers specialized in the education of DHH learners (Hyde and Power, 2003; Luckner, 2006). These teachers typically receive specialized professional preparation focused on addressing the diverse educational needs of these students across different developmental stages, from early childhood through secondary education (Luckner, 2010; Luckner and Ayantoye, 2013).

The support provided by specialized teachers varies according to students' educational and social needs; however, it generally focuses on developing auditory, linguistic, academic, and social competencies (Luckner, 2010). These teachers provide both direct support to DHH students and collaborative support to general education teachers and other support personnel. Given the broad impact of hearing loss on child development, this support often continues throughout students' educational trajectories, resulting in long-term teacher-student relationships (Luckner, 2011). Such sustained relationships provide teachers with deeper insights into how hearing loss influences linguistic, academic, social, and emotional development across developmental stages (Norman and Jamieson, 2015).

Within this context, social and emotional performance (SEP) is considered a key concept for understanding how social and emotional competencies develop and how they can be effectively supported (Norman and Jamieson, 2015). This concept refers to a range of abilities related to recognizing and managing emotions, demonstrating empathy and care for others, building positive relationships, making responsible decisions, and responding constructively to challenging situations (Anthony et al., 2020; Collaborative for Academic, 2015; Zins et al., 2004). Social and emotional performance is also associated with competencies such as self-awareness, self-management, and social awareness (Camras and Halberstadt, 2017; Chen and French, 2008). Difficulties in social and emotional performance arise when deficits in age-appropriate social and emotional competencies hinder individuals' wellbeing (Carter et al., 2004). These challenges may manifest in observable behaviors such as attention difficulties, antisocial behavior, anxiety, and limited social interaction (Scholte and van Der Ploeg, 2017).

A substantial body of research has demonstrated that social and emotional competencies—including self-regulation, self-discipline, and interpersonal skills—play a critical role in academic achievement, career success, and overall psychological wellbeing (Kautz et al., 2014). Scholars have also emphasized that academic success depends not only on cognitive abilities but also on social and emotional factors such as motivation, self-regulation, and positive attitudes toward learning (Al-zahrani et al., 2019; Heckman and Kautz, 2012). Therefore, assessing children's social and emotional competencies is essential for the early identification of social and emotional difficulties and the development of appropriate interventions, particularly for DHH students whose communication challenges alone do not fully explain the social and emotional difficulties they may encounter (Smit et al., 2023).

Therefore, social and emotional learning further focuses on developing essential life skills that enable children to manage themselves, interact effectively with others, and navigate everyday challenges (Walberg et al., 2004). Consequently, numerous educational programs have been developed to promote social and emotional learning in schools, with teachers serving as the primary agents responsible for implementing these programs. This underscores the importance of collaborative efforts among teachers, educational staff, and families to support the development of social and emotional competencies among DHH students, thereby enhancing their social integration and academic success (Herzig and Leannah, 2022).

In light of the above, it becomes essential to examine teachers' perceptions of the social and emotional performance of DHH students in inclusive schools, as these perceptions play an important role in shaping future educational practices and strategies that support students' social and emotional development.

## 2 Research problem

Social and emotional performance is frequently regarded as a key factor influencing individuals' psychological and social wellbeing, as well as the nature of their daily interactions within their social environments. This type of performance is associated with a range of abilities and behaviors, including social participation, attention and concentration, empathy, understanding others' perspectives, and the expression of different emotions, among other important social competencies (Chernyshenko et al., 2018). From this perspective, the early identification and diagnosis of social and emotional difficulties are critically important, as they enable the implementation of appropriate interventions and the provision of effective support systems, thereby reducing potential negative effects on individuals' social lives and psychological wellbeing (Kawara and Michaela, 2025).

Within the educational context of DHH students, the long-term educational relationship between teacher and student acquires particular importance. Over time, this relationship evolves into one based on trust and continuity of support within the educational environment. Teachers are often the individuals most familiar with the impact of hearing loss on different aspects of students' development, including its implications for their social and emotional needs. Consequently, teachers of DHH students occupy a unique position that enables them to provide direct support and valuable insights into how students' social and emotional development can be fostered (Norman and Jamieson, 2015). Moreover, teachers' beliefs play a central role in shaping their instructional practices. Pajares (1992) argues that teachers who place importance on developing students' social and emotional competencies alongside academic achievement are more likely to integrate social and emotional learning into their daily classroom practices. Because teachers' beliefs directly influence the educational decisions and instructional strategies they adopt, examining these beliefs can contribute to a deeper understanding of how teachers support their students' social and emotional development.

In recent years, research on DHH individuals has expanded beyond auditory and linguistic aspects to include broader domains such as academic performance, cognition, health-related quality of life, mental health, and the social and emotional performance of DHH children and adolescents. Nevertheless, studies addressing this aspect remain relatively limited, particularly those that consider the personal and social backgrounds of these students (Kawara and Michaela, 2025).

Research also indicates that a wide range of social and emotional learning programs have been implemented with hearing students and students with disabilities across various educational stages. These programs have been shown to improve students' social and emotional skills, strengthen positive attitudes toward themselves, others, and school, and enhance both social behavior and academic performance (Dymnicki et al., 2012; Osher et al., 2016; Sklad et al., 2012). Despite these positive outcomes, however, available evidence regarding the effectiveness of social and emotional performance interventions specifically designed for DHH individuals remains limited. There is a lack of sufficient high-quality research to identify evidence-based interventions in this field, which poses challenges for parents, teachers, early intervention specialists, and educational administrators seeking to support this group of students (Luckner and Movahedazarhouligh, 2018).

More specifically, the literature indicates that research examining teachers' perceptions of the social and emotional performance of DHH students remains limited. Previous studies have not sufficiently explored teachers' readiness to support students' social and emotional needs or the types of practices teachers employ to identify and address these needs within inclusive educational settings (Norman and Jamieson, 2015). In this context, Brackett et al. (2012) suggest that examining teachers' beliefs regarding social and emotional performance can provide an important entry point for understanding how teachers support students' social and emotional development, particularly in contexts where teachers rely more heavily on interpersonal relationships and individual interactions rather than formal social and emotional learning programs.

Accordingly, examining teachers' perceptions of the social and emotional performance of DHH students may contribute to filling an important knowledge gap in the educational literature. It may also provide deeper insights into teachers' roles in supporting the psychological and social wellbeing of DHH students within inclusive schools. Furthermore, the findings of this study may offer practical insights that contribute to improving teacher preparation and professional development programs, thereby enhancing teachers' ability to support the social and emotional needs of their students. in light of the above, the present study seeks to answer the following main research question:

**What are teachers' perceptions of the social and emotional performance of DHH students in inclusive schools?**

## 3 Research questions

The study seeks to answer the following research questions, which are organized according to the type of data and analytical purpose:

**Quantitative Questions**

1. What is the level of social and emotional performance of DHH students in inclusive schools from teachers' perspectives?
2. Are there statistically significant differences at the level of (α = 0.05) in teachers' scores on the Social and Emotional Performance Scale attributable to the following variables: Academic qualification (Special Education Bachelor's degree, General Education Bachelor's degree, Master's degree, Other); Years of teaching experience (less than one year, 1–5 years, more than 5 years); Educational stage (early childhood, primary, intermediate, secondary); Teaching category (deaf students, hard-of-hearing students, both DHH students)?

**Qualitative Questions**

3. What factors influence the social and emotional performance of DHH students from teachers' perspectives?
4. What strategies do teachers propose to enhance the social and emotional performance of DHH students?

**Integration Question (Mixed Methods)**

5. How do the contextual factors identified through qualitative analysis influence the levels of social and emotional performance measured quantitatively among DHH students?

## 4 Theoretical framework and previous studies

Individuals, in general, need to develop the ability to understand and appropriately manage their emotions, interact positively with others, and exercise sound judgment in various life situations. In this context, DHH individuals are considered a group at greater risk of not acquiring age-appropriate social and emotional skills compared with their hearing peers (Luckner and Movahedazarhouligh, 2018). Success in school and in life does not depend solely on mastery of academic content; rather, it also requires a set of social and emotional competencies that include the ability to understand and regulate emotions, interact positively with others, demonstrate responsibility, show concern for others, and make sound and healthy decisions (Melnick et al., 2017). Numerous studies have indicated that mastery of these competencies—often referred to as social and emotional performance—is associated with higher levels of wellbeing and improved academic outcomes. Conversely, deficiencies in these skills may lead to a range of personal, social, academic, and economic challenges (Durlak et al., 2011; Guerra and Bradshaw, 2008).

Research literature further suggests that individuals with strong social and emotional competencies are more likely to achieve success across various areas of life compared with those who lack such competencies. Conversely, low levels of social and emotional competence may increase the likelihood of social rejection and heighten vulnerability to mental health problems that may persist into adulthood. With respect to DHH individuals, research has shown that they may encounter difficulties in developing social and emotional skills, such as recognizing and managing emotions, setting and pursuing positive goals, understanding others' perspectives, and building and maintaining positive relationships. These challenges are further compounded by the limited body of research addressing social and emotional development among DHH individuals, which could otherwise inform educational practices implemented by caregivers and teachers (Luckner and Movahedazarhouligh, 2018).

Studies examining the roles and responsibilities of teachers of DHH students in educational and training settings have emphasized the significant role these teachers play in supporting their students' social and emotional development. Such support typically involves fostering self-advocacy skills, teaching strategies for coping and personal independence, guiding students in navigating social situations, and helping them develop social competencies and establish positive relationships with peers (Antia et al., 2011; Foster and Cue, 2009). In this regard, the professional context in which teachers of DHH students operate differs in several important ways from that of general education teachers. On the one hand, these teachers often work with students individually or in small groups, which may make students' social and emotional needs more visible compared with those in larger classroom settings. On the other hand, teachers' beliefs regarding the importance of teaching social and emotional learning and their readiness to assume this educational role remain insufficiently explored in the literature. Similarly, the importance of professional development opportunities that could enhance teachers' competence in teaching social and emotional learning remains inadequately defined. Furthermore, many teachers of DHH students work across multiple schools, meaning they interact with diverse school cultures that vary in the extent to which they support or value efforts to promote social and emotional learning among DHH students (Norman and Jamieson, 2015).

Regarding the effectiveness of social and emotional learning programs for DHH students, a meta-analysis of 213 comprehensive programs in this field revealed that students who participated in these programs demonstrated significant improvements in their attitudes toward themselves and others, as well as improvements in positive social behaviors and stronger engagement with school compared with students in control groups. These findings suggest that developing social and emotional competencies is a fundamental component of the holistic development of DHH students, given its long-term impact on their academic, social, and emotional development (Durlak et al., 2011). It is also noteworthy that the Promoting Alternative Thinking Strategies (PATHS) program, developed by Greenberg and Kusché (1998), is among the early programs designed to support the social and emotional development of DHH children. This program was originally designed as a school-based intervention aimed at promoting effective communication skills, including problem solving, emotional awareness, emotional regulation, and self-control. Subsequent studies have demonstrated the long-term effectiveness of social and emotional learning programs not only in general education classrooms (Kelly et al., 2004), but also in special education settings (Kam et al., 2004) and classrooms serving DHH students (Greenberg and Kusché, 1998).

Overall, the literature indicates that social and emotional performance represents an essential dimension of the holistic development of DHH students, given its direct influence on their psychological and social wellbeing as well as their academic adjustment across different educational stages.

## 5 Materials and methods

### 5.1 Methodology

A sequential explanatory research design, integrating both quantitative and qualitative approaches, was considered the most appropriate for the purposes of this study. Specifically, the nature of the research questions required moving from initial quantitative findings to a more in-depth qualitative exploration in the second phase.

This design is widely used in the social, behavioral, and health sciences, as it allows researchers to collect both quantitative and qualitative data, integrate the two forms of evidence, and generate interpretations that draw upon the strengths of each data source to better understand the research problem or phenomenon under investigation (Creswell, 2015). The design consists of two phases: an initial phase employing quantitative methods to obtain preliminary data, followed by a second phase utilizing qualitative methods to provide deeper explanations of the results derived from the first phase. The two methodologies are integrated during the participant selection stage and again during the interpretation of the findings (Creswell, 2015).

Mixed-methods research designs may take various forms and are selected to address three key aspects of a study: sequence, priority (weight), and integration. The sequence dimension determines when quantitative and qualitative data are collected and utilized within the study. The priority dimension refers to the relative importance assigned to quantitative and qualitative data in addressing the research questions. The integration dimension concerns where and how quantitative and qualitative data are combined (Creswell, 2015). Based on these design characteristics and the objectives of the study, the sequential explanatory design was deemed the most suitable for the present research, with particular emphasis on the research questions as the primary guide for these methodological decisions. Because the purpose of the quantitative data was to provide a broad overview of the study context, facilitate participant selection for the qualitative phase, and inform the development of follow-up interview questions, quantitative data were collected first (Creswell and Plano Clark, 2011). Given that answering the primary research questions required a deeper understanding of participants' perspectives and perceptions regarding the phenomenon under investigation, priority was assigned to the qualitative data. Ultimately, the quantitative and qualitative data were integrated during the interpretation stage after addressing the research questions.

**Figure 1:**
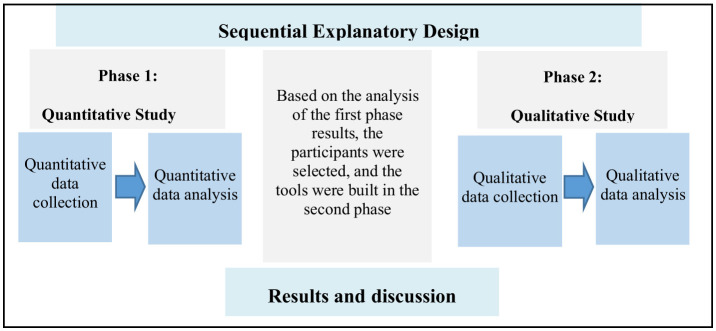
The study design scheme.

Accordingly, the study was conducted in two phases. First, the level of social and emotional performance was identified through the quantitative phase. Second, the main factors influencing this performance and the key strategies proposed to enhance it were identified through the qualitative phase. In this way, quantitative and qualitative data were collected and analyzed to interpret the study findings. Figure 1 illustrates the overall research design.

### 5.2 Participants

The study population consisted of all teachers of DHH students working in inclusive schools in the Kingdom of Saudi Arabia. The questionnaire was distributed both electronically and in person to maximize coverage of the target population. Specifically, it was administered manually to female teachers in Al-Ahsa Governorate due to the researcher's direct access to participants in this region, while electronic distribution was used to reach female teachers in other regions across the Kingdom of Saudi Arabia. This process resulted in 102 valid responses, which is considered adequate for quantitative analysis given the nature of the population and the challenges associated with accessing it. Although the sampling approach was primarily based on accessibility, efforts were made to ensure reasonable representation of the target population. It is important to note that the number of female teachers of DHH students in Al-Ahsa Governorate is (42) teachers, according to official statistics from the Ministry of Education for the academic year 2026. This region represents the context in which the instrument was administered manually. In contrast, the questionnaire was distributed electronically across other regions of the Kingdom through multiple educational administrations and professional channels. Due to the decentralized nature of this distribution and the diversity of platforms used, it was not possible to determine the exact total number of teachers who received or accessed the scale.

From this group, a purposive sample of 15 female teachers of DHH students was selected for the qualitative phase. Participants were selected based on the sensitivity of the research topic and the difficulty of conducting in-depth qualitative research with the entire target population. The selection of participants was based on several criteria: (1) Participants were teachers of DHH students working in inclusive schools in Saudi Arabia. (2) Participants' characteristics were aligned with the objectives of the study. (3) Gender considerations were taken into account; female participants were selected due to the practicality of conducting face-to-face interviews with them, as conducting interviews with male teachers is generally restricted in the Saudi educational context. (4) Given the sensitivity of the population under study, limiting the qualitative sample size allowed for a more in-depth investigation while minimizing participant burden and ethical risks.

Data collection continued until no new significant themes emerged, indicating the achievement of thematic saturation, which represents a key criterion for determining adequacy in qualitative data collection (Cunningham and Carmichael, 2017). It is acknowledged that the selection of the study site and participants' demographic characteristics may limit the generalizability of the findings. Therefore, these factors were considered during the interpretation of the results, and the findings were presented as representative of the available context rather than all teachers of DHH students at the national level. Tables 1, 2 present the demographic and personal characteristics of the participants.

**Table 1:** Demographic characteristics of participants in the quantitative study.

Variable	Category	Frequency	Percentage
Educational qualification	Special education bachelor's	67	65.7%
	General education bachelor's	17	16.6%
	Master's degree	16	15.7%
	Other	2	2%
Educational stage	Early childhood	19	18.6%
	Primary school	34	33.3%
	Intermediate school	23	22.6%
	Secondary school	26	25.5%
Years of teaching experience	Less than 1 year	6	5.9%
	1–5 years	15	14.7%
	More than 5 years	81	79.4%
Teaching category	Deaf students	16	15.7%
	Hard-of-hearing students	41	40.2%
	DHH students	45	44.1%

**Table 2:** Demographic characteristics of participants in the qualitative study.

Variable	Category	Frequency	Percentage
Educational qualification	Special education bachelor's	7	46.7%
	Master's degree	7	46.7%
	Other	1	6.6%
Years of experience teaching DHH students	Less than 1 year	1	6.7%
	1–5 years	2	13.3%
	More than 5 years	12	80.0%
Educational stage	Early childhood	2	13.3%
	Primary	2	13.3%
	Intermediate	3	20.0%
	Secondary	8	53.4%
Teaching category	Deaf students	1	6.7%
	Hard-of-hearing students	7	46.7%
	DHH students	7	46.7%

### 5.3 Research instruments

The researcher employed two main instruments in this study. Both instruments were designed to align with the objectives of the study and the characteristics of the target population, as described below.

#### 5.3.1 Social and emotional performance scale for DHH students (developed by the researcher)

The Social and Emotional Performance Scale was used as the primary quantitative instrument in this study. The scale was developed by the researcher to assess the level of social and emotional performance among DHH students from the perspectives of their teachers. The scale was constructed after reviewing previously developed instruments designed for similar purposes and examining relevant literature (Norman and Jamieson, 2015; Anthony et al., 2020; Antia et al., 2011; Batten et al., 2014).

##### 5.3.1.1 Psychometric properties of the scale

At the outset, it should be noted that all psychometric analyses of the instrument—including the Kaiser-Meyer-Olkin (KMO) measure of sampling adequacy, Bartlett's test of sphericity, exploratory factor analysis (EFA), internal consistency validity, and Cronbach's alpha reliability coefficients—were conducted using the same primary quantitative sample of the study (*N* = 102) of Teachers. A detailed explanation of each is provided below:

**First: face and content validity:** To ensure content validity, the preliminary version of the scale was reviewed by five experts in special education and psychology. The experts evaluated the relevance, clarity, and representativeness of the scale items for the target population. Based on their feedback, several modifications were made, which enhanced both the face validity and content validity of the instrument.

**Second: construct validity:** The results of the exploratory factor analysis (EFA) indicated a clear two-factor structure consistent with the theoretical framework of the scale. Items designed to measure social performance loaded on the first factor, whereas emotional performance items loaded on the second factor. The factor loadings for each item were examined to determine the extent to which each item is associated with its corresponding dimension, and all retained items demonstrated acceptable factor loadings on their respective factors. Based on these results, items with weak or ambiguous loadings were removed in accordance with established statistical criteria. As a result, the final version of the scale was refined to include a total of) 22(items. This alignment between the hypothesized theoretical structure and the empirically derived factor structure provides supporting evidence for the construct validity of the instrument (see Table 3).

**Table 3:** X. Factor loadings of the social and emotional performance scale.

Item code	Item statement	Factor loading	Factor
Factor 1: social performance (15 items, α = 0.904)			
A1	The student interacts positively with classmates in the classroom	0.682	Social
A2	The student participates in group activities at school	0.705	Social
A3	The student is able to form friendships with peers	0.789	Social
A4	The student demonstrates the ability to cooperate with others	0.635	Social
A5	The student expresses feelings in front of others	0.621	Social
A6	The student shows initiative in direct communication situations	0.689	Social
A7	The student takes the initiative to help classmates when needed	0.750	Social
A8	The student maintains positive relationships with friends	0.695	Social
A9	The student demonstrates the ability to resolve simple conflicts	0.402	Social
A10	The student is socially accepted among peers	0.587	Social
A11	The student demonstrates socially responsible behavior	0.676	Social
A12	The student possesses positive social interaction skills	0.590	Social
A13	The student responds positively to emotional support from the teacher	0.485	Social
A14	The student is able to express emotions using sign language or alternative means	0.464	Social
A15	The student easily expresses feelings of joy upon success	0.563	Social
Factor 2: emotional performance (7 items, α = 0.771)			
A16	The student avoids getting into problems with classmates	0.446	Emotional
A17	The student demonstrates the ability to regulate emotions	0.560	Emotional
A18	The student demonstrates emotional stability under academic pressure	0.712	Emotional
A19	The student deals calmly with difficult situations	0.754	Emotional
A20	The student expresses feelings of joy and happiness naturally	0.619	Emotional
A21	The student shows anxiety or tension in new situations	0.596	Emotional
A22	The student is able to overcome feelings of frustration after failure	0.677	Emotional

**Third: suitability of the data for factor analysis:** The Kaiser-Meyer-Olkin (KMO) measure of sampling adequacy was 0.852, indicating a high level of adequacy. Bartlett's test of sphericity was statistically significant (*p* < 0.001), confirming that the data were appropriate for factor analysis.

**Fourth: internal consistency validity:** To assess the internal consistency validity of the scale, Pearson correlation coefficients were calculated between each item and its corresponding dimension, as well as with the overall scale score. The results demonstrated that all items showed statistically significant correlations at the 0.01 level, indicating satisfactory internal consistency. Specifically, the fifteen items of the social dimension were significantly correlated with both their respective dimension and the overall scale, with coefficients ranging from 0.404 to 0.773. Similarly, the seven items of the emotional dimension exhibited significant correlations with their dimension and the total scale, with coefficients ranging from 0.329 to 0.649.

In addition, the relationship between the two dimensions was found to be moderately positive and statistically significant (*r* = 0.438, *p* < 0.01), suggesting that while the dimensions are related, they capture distinct aspects of the construct. This is further supported by the strong correlations observed between each dimension and the overall scale, where the social dimension showed a very strong association (*r* = 0.947, *p* < 0.01) and the emotional dimension demonstrated a strong association (*r* = 0.703, *p* < 0.01). Collectively, these findings provide evidence of the internal consistency and structural validity of the instrument.

**Fifth: reliability of the instrument:** The overall Cronbach's alpha coefficient for the scale was (0,900) indicating a high level of internal consistency. At the subscale level, the reliability coefficient for the social performance dimension was (0,904), while the emotional performance dimension yielded a coefficient of (0,771), The relatively lower Cronbach's alpha coefficient for the emotional dimension, compared to the social dimension, can be attributed to the smaller number of items (7 items vs. 15), as Cronbach's alpha is directly influenced by the number of items.reflecting good and acceptable levels of reliability.

Based on the above, it can be concluded that the instrument demonstrates satisfactory levels of validity and reliability, supporting its use as a robust tool for assessing the social and emotional performance of deaf and hard-of-hearing students within the context of the present study.

##### 5.3.1.2 Scale structure

Based on the procedures described above, the final version of the scale consisted of two sections: Section One: Demographic information including four variables: educational qualification, years of teaching experience, educational stage, and teaching category. Section Two: The main section of the scale consisting of 22 items, divided into two dimensions: Social Performance (items 1–15), Emotional Performance (items 16–22). Each item was rated using a five-point Likert scale: (1 = Never, 2 = Rarely, 3 = Sometimes, 4 = Often, 5 = Always) (see Appendix A).

#### 5.3.2 Individual interviews

A semi-structured individual interview served as the primary instrument for collecting qualitative data in this study. Semi-structured interviews were used to obtain rich and detailed data from participants while allowing flexibility in modifying questions when necessary (Abu 'Allam, 2007). The interviews included main and follow-up questions focusing on participants' experiences and perceptions regarding the following topics: Factors influencing the social and emotional performance of DHH students, Strategies proposed by teachers to enhance the social and emotional performance of DHH student (see Appendix B). Semi-structured interviews were conducted with 15 teachers who had previously completed the scale. Each interview lasted approximately 50–60 min. Field notes were recorded during and immediately after each interview to document contextual information and initial analytical reflections. Each session was audio-recorded with participants' consent and subsequently transcribed verbatim for analysis.

### 5.4 Data collection

After obtaining the required ethical and administrative approvals, the researcher distributed the study instruments to the target sample. The scale was initially distributed to all teachers of DHH students in Saudi Arabia. A total of 102 responses were received, while some teachers declined participation. After reviewing and analyzing the responses, teachers were purposively selected according to the predetermined criteria to participate in the interview phase. During data collection, efforts were made to create a supportive educational environment that promoted psychological comfort and encouraged honest and accurate responses. The qualitative phase followed a series of structured procedures. First, the interview questions were reviewed and validated by specialists in qualitative research. Data were then collected and subsequently analyzed. To ensure participants' privacy and comfort during the interviews, the sessions were conducted face-to-face in a quiet setting, using a semi-structured interview guide to facilitate open and accurate responses. Data collection continued until no new significant themes emerged, indicating that thematic saturation had been reached. This strategy strengthened the explanatory function of the sequential design, allowing qualitative data to directly interpret and clarify key quantitative findings.

### 5.5 Data analysis

Quantitative data were analyzed using SPSS (Version 20). The analysis included the following steps: (1) Calculating means and standard deviations to determine teachers' overall scores on the Social and Emotional Performance Scale and its dimensions. (2) Conducting the Kruskal-Wallis test for independent samples to examine differences in teachers' scores according to the study variables: educational qualification, years of teaching experience, educational stage, and teaching category.

For the qualitative data, thematic analysis (TA) was used to identify, analyze, and present emerging themes. This approach was considered appropriate because it allowed the researcher to interpret qualitative data through an inductive process, deriving themes directly from the data rather than relying on predetermined categories. The analysis followed the sequential procedures proposed by Braun and Clarke (2006), beginning with familiarization with the data, followed by generating initial codes, identifying and reviewing themes, and finally producing the report and linking findings with relevant literature. Thematic analysis is widely recognized as one of the most commonly used approaches in qualitative data analysis, whereby the researcher identifies categories based on the data and their relationship to the research focus, and in some cases to the research questions (Bryman, 2008). Accordingly, this study was grounded in a clear methodological and philosophical alignment between epistemological assumptions, research methodology, and the interpretation of results.

### 5.6 Trustworthiness and credibility of qualitative data

The objectivity and credibility of the qualitative findings were ensured by applying several procedures recommended by Abu 'Allam (2007) and Al-Abdulkareem (2012), including the following: (1) Triangulation: Data were collected from two sources—the Social and Emotional Performance Scale and individual interviews—to enhance validity and reduce potential bias. (2) Expert Review: Three faculty members from Saudi universities specializing in education reviewed the interview questions. Based on their feedback, several modifications were made, including deleting some questions, rephrasing others, and adding more detailed questions. (3) Systematic Coding: The researcher maintained coded analytical notes throughout the analysis process to document emerging ideas and ensure systematic data organization. (4) Member Checking: To ensure the accuracy of the data, participants were provided with full written transcripts of their interviews and were asked to review them and confirm their accuracy.

## 6 Results

### 6.1 Results for first research question

To answer this question, means and standard deviations were calculated for teachers' total scores on the Social and Emotional Performance Scale for DHH Students. The results are presented in Table 4.

**Table 4:** Means and standard deviations of teachers' total scores on the social and emotional performance scale for DHH students.

No.	Item	Mean	SD
1	The student interacts positively with classmates in the classroom.	3.902	0.8023
2	The student participates in group activities at school.	3.578	0.9793
3	The student is able to form friendships with peers.	3.902	0.9174
4	The student demonstrates the ability to cooperate with others.	4.049	0.9051
5	The student expresses feelings in front of others.	3.421	0.9589
6	The student shows initiative in situations requiring direct communication.	3.235	1.0164
7	The student takes the initiative to help classmates when needed.	3.813	1.0691
8	The student maintains positive relationships with friends.	3.872	0.7793
9	The student shows the ability to resolve simple conflicts with classmates.	3.137	1.0903
10	The student is socially accepted among peers.	3.705	0.8854
11	The student demonstrates socially responsible behavior.	3.676	0.8578
12	The student possesses positive social interaction skills.	3.352	0.9189
13	The student responds positively to emotional support from the teacher.	4.333	0.6941
14	The student is able to express emotions using sign language or alternative means.	4.137	0.8447
15	The student can easily express feelings of joy when succeeding in academic tasks.	4.245	0.7889
16	The student avoids getting into problems with classmates.	3.519	0.9823
17	The student demonstrates the ability to regulate emotions in different situations.	3.058	0.8422
18	The student demonstrates emotional stability when facing academic pressure.	3.147	0.8943
19	The student deals calmly with difficult situations.	3.225	0.9000
20	The student expresses feelings of joy and happiness naturally.	4.127	0.8165
21	The student shows anxiety or tension in new situations.	3.460	0.9613
22	The student is able to overcome feelings of frustration after failure.	3.352	0.7916
Total		3.64	0.477

The results indicate that the overall mean score of the scale was (M = 3.64, SD = 0.477), which falls within the moderate level according to the adopted classification. This suggests that deaf and hard-of-hearing (DHH) students demonstrate an acceptable level of social and emotional performance; however, there remains a need for further enhancement through targeted educational and support interventions. The relatively low standard deviation also indicates a reasonable level of agreement among teachers in their evaluations. Table 5 presents the classification used to determine the level of rating.

**Table 5:** Mean score ranges and corresponding rating levels.

Mean score range	Rating level
1.00–2.33	Low
2.34–3.67	Moderate
3.68–5.00	High

With regard to individual items, the highest mean score was recorded for Item (13), “The student responds positively to emotional support from the teacher” (M = 4.33), followed by Item (15), “The student can easily express feelings of joy when succeeding in academic tasks” (M = 4.25), and Item (14), “The student is able to express emotions using sign language or alternative means” (M = 4.14). These results indicate that DHH students demonstrate strong abilities in responding to emotional support and expressing positive emotions, particularly within supportive and structured school environments. They also highlight the important role of teachers and effective communication methods in enhancing students' emotional wellbeing and social interaction. Similarly, relatively high mean scores were observed for Item (4), “The student demonstrates the ability to cooperate with others” (M = 4.05), and Items (1) and (3), related to positive interaction and friendship formation (M = 3.90), reflecting students' ability to engage socially with peers.

In contrast, the lowest mean scores were observed for Item (17), “The student demonstrates the ability to regulate emotions in different situations” (M = 3.06), followed by Item (9), “The student shows the ability to resolve simple conflicts with classmates” (M = 3.14), and Item (18), “The student demonstrates emotional stability when facing academic pressure” (M = 3.15). These findings suggest that emotional regulation, conflict resolution, and coping with academic pressure are comparatively less developed among DHH students.

Overall, the results reveal that students perform better in socially supported and emotionally positive contexts—particularly those involving teacher support and successful experiences—while facing relatively greater challenges in managing emotions in complex or stressful situations. This highlights the need for targeted interventions focusing on emotional regulation, adaptive coping strategies, and social problem-solving skills to further enhance students' social and emotional performance.

### 6.2 Results for second research question

To answer this question, the Kruskal-Wallis test was used to identify differences in teachers' views regarding the level of social and emotional performance of DHH students according to the study variables: educational qualification, years of experience, educational stage, and teaching category. In addition, epsilon-squared (ε^2^) was calculated as a measure of effect size using the following formula: ε^2^ = (H – k + 1) / (n – k). The values were interpreted as follows: ε^2^ < 0.01 indicates a very small effect, 0.01–0.06 indicates a small effect, 0.06–0.14 indicates a moderate effect, and ε^2^ > 0.14 indicates a large effect. The results are presented in Tables 6–9.

**Table 6:** Kruskal-Wallis test for differences in teachers' views according to educational qualification.

Groups	*n*	Mean rank	Kruskal-Wallis value	Significance Level	Effect size ε^2^	Effect size level
Special education bachelor's	67	48.92	7.78	0.051	0.049	Small effect
General education bachelor's	17	65.88				
Master's degree	16	43.31				
Other	2	81.25				

**Table 7:** Kruskal-Wallis test for differences in teachers' views according to years of experience.

Groups	*n*	Mean rank	Kruskal-Wallis value	Significance level	Effect size ε^2^	Effect size level
Less than 1 year	6	42.67	0.602	0.740	0.00	Very small effect
1–5 years	15	53.33				
More than 5 years	81	51.81				

**Table 8:** Kruskal-Wallis test for differences in teachers' views according to educational stage.

Groups	*n*	Mean rank	Kruskal-Wallis value	Significance level	Effect size ε^2^	Effect size level
Early childhood	19	43.39	3.17	0.366	0.002	Very small effect
Primary school	34	52.29				
Intermediate school	23	48.89				
Secondary school	26	58.69				

**Table 9:** Kruskal-Wallis test for differences in teachers' views according to teaching category.

Groups	*n*	Mean rank	Kruskal-Wallis value	Significance level	Effect size ε^2^	Effect size level
Deaf students	16	66.00	4.589	0.101	0.026	Small effect
Hard-of-hearing students	41	48.28				
DHH students	45	49.28				

#### 6.2.1 Educational qualification

As shown in Table 6, the significance level was (*p* = 0.051), indicating that there were no statistically significant differences in teachers' views regarding the level of social and emotional performance of DHH students attributable to educational qualification at the conventional level (α = 0.05). However, this value is very close to the significance threshold, suggesting a marginal effect and indicating the possibility of differences that may become statistically significant with a larger or more balanced sample.

##### 6.2.1.1 *Post-hoc* pairwise comparisons for academic qualification

Given that the significance level for the academic qualification variable was (*p* = 0.051), which is very close to the threshold of statistical significance, *post-hoc* pairwise comparisons were conducted among all categories of academic qualification using the Mann-Whitney *U* test with Bonferroni correction to control for Type I error (adjusted α = 0.05 ÷ 6 = 0.008). The results are presented in Table 10.

**Table 10:** *Post-hoc* pairwise comparisons for academic qualification (Mann-Whitney *U* Test with Bonferroni correction).

Comparison	*U*	*p*	Adjusted *p*	*r*	Significance
Special education bachelor's vs. general education bachelor's	445.5	0.169	1.000	0.150	Not significant
Special education bachelor's vs. master's degree	616.5	0.356	1.000	0.101	Not significant
Special education bachelor's vs. other	27.5	0.163	0.977	0.168	Not significant
General education bachelor's vs. master's degree	192.0	0.045	0.272	0.348	Marginal trend
General education bachelor's vs. other	9.5	0.351	1.000	0.214	Not significant
Master's degree vs. other	3.0	0.079	0.472	0.415	Not significant

It is evident from Table 10 that no statistically significant differences were found between any pair of academic qualification categories after applying the Bonferroni correction (α = 0.008). However, the comparison between General Education Bachelor's and Master's Degree showed a marginal trend (*p* = 0.045, adjusted *p* = 0.272, *r* = 0.348), which may warrant further investigation in studies with larger sample sizes. This finding is consistent with the non-significant overall result of the Kruskal-Wallis test.

#### 6.2.2 Years of experience

As shown in Table 7, the significance level was (0.740), indicating that there were no statistically significant differences in teachers' views regarding the level of social and emotional performance of DHH students attributable to years of experience.

#### 6.2.3 Educational stage

As shown in Table 8, the significance level was (0.366), indicating that there were no statistically significant differences in teachers' views regarding the level of social and emotional performance of DHH students attributable to educational stage.

#### 6.2.4 Teaching category

As shown in Table 9, the significance level was (0.101), indicating that there were no statistically significant differences in teachers' views regarding the level of social and emotional performance of DHH students attributable to teaching category.

This finding is consistent with the overall pattern observed across the examined variables, as the Kruskal–Wallis tests did not reveal any statistically significant differences. The effect sizes (ε^2^) were small or negligible across all comparisons, including academic qualification (ε^2^ = 0.049), teaching category (ε^2^ = 0.026), educational stage (ε^2^ = 0.002), and years of experience (ε^2^ ≈ 0.00), indicating limited practical significance of the observed differences.

### 6.3 Results for third research question

To answer the third research question, the researcher used thematic analysis (TA) to analyze participants' responses. The analysis identified a set of major factors influencing the social and emotional performance of DHH students in inclusive schools. These factors were classified into three interrelated main themes, as follows:

#### 6.3.1 The role of family, peers, and teachers in enhancing social and emotional performance

Participants explained that the family plays a central and essential role in enhancing the self-confidence of DHH students through unconditional acceptance, continuous emotional support, involving the student in family decision-making, and providing opportunities to assume responsibilities appropriate to their abilities. One participant expressed this by stating: “*The family enhances social and emotional performance by providing love and emotional containment, reinforcing small achievements, allowing the student to participate in family decisions, and giving them various responsibilities that suit their abilities and encourage them, and all of this is reflected in the student's self-confidence*.” (P14). Some participants also noted that having deaf family members positively contributes to students' psychological and social adjustment. As one participant stated: “*A supportive family from an early age, unconditional acceptance, and providing an environment full of love and security, as well as having deaf family members, greatly affects DHH individuals psychologically and socially.”* (P4). Participants further emphasized the importance of treating the student as a capable individual, without excessive protection or pity. One participant stated: “*They should be treated like hearing students and given tasks and responsibilities from an early age.”* (P3).

In the same vein, peers emerged as an important factor in fostering students' sense of belonging and self-worth through shared activities, learning basic sign language, and engaging in interactions based on mutual respect. One participant explained: “*When peers learn some signs and interact respectfully, the student feels that they are a natural member of the group.”* (P10). Another participant highlighted the role of peers in developing students' social interaction skills: “*Peers' role comes through joint activities in learning or play, so the student learns patience in interaction and mutual respect, and is therefore supported in situations that require help.”* (P7)

In addition, participants highlighted the role of the teacher in supporting social and emotional performance. Building a relationship based on trust and emotional support was described as an essential gateway to emotional expression. One participant stated: “*Trust and feeling comfortable with the teacher are the main reasons for expressing oneself freely; however, the problem is that this is often a voluntary practice by the teacher, as teachers do not receive sufficient training to provide these services to students but rather rely on their personal experience and professional intuition.”* (P3). Participants also emphasized the importance of one-to-one communication between the student and the hearing-inclusion teacher, as well as the teacher's role as a model in regulating emotions and behaviors, which positively influences students.

#### 6.3.2 Communication methods used

The qualitative findings showed that the communication methods used by DHH students represent a significant factor influencing their level of social and emotional performance. Patterns of emotional expression and social interaction were found to vary according to the communication mode used, whether sign language, spoken language, or total communication. Users of sign language, for example, may differ in how they express feelings and emotions compared with users of spoken language or total communication. Participants stressed the importance of providing students with a safe space to express their feelings using methods that align with their characteristics. One participant stated: “*Communication methods help hard-of-hearing students by talking with them and giving them a comfortable space to express themselves and their feelings, through communication methods that suit their characteristics, whether sign language or others.”* (P12). Other participants also emphasized the effectiveness of using total communication, encouraging safe emotional expression, training students in simple emotional vocabulary, and using modeling, role play, and storytelling, while respecting personal space and maintaining calm eye contact. These strategies were seen as facilitating students' communication with others. One participant stated: “*Using the appropriate communication method makes it easier for them to communicate with their peers and reduces their sense of isolation.”* (P10). These findings suggest that the diversity and flexible use of communication methods contribute to improving social interaction and reducing feelings of isolation, thereby enhancing students' emotional and social stability.

#### 6.3.3 Educational environment and assistive resources

Participants indicated that a supportive educational environment plays a central role in enhancing the social and emotional performance of DHH students, particularly in inclusive classrooms, by providing opportunities for peer interaction, counseling sessions, and relationships with students facing similar challenges. One participant stated: “*Inclusive classrooms with hearing students play a major role in their social and emotional performance. This happens through counseling sessions with peers who have similar challenges, which reduces their negative feelings.”* (P5). Participants also emphasized that creating a school environment based on acceptance and respect contributes to enhancing students' sense of safety and self-confidence, in addition to classroom practices such as rotating seating arrangements and encouraging hearing classmates to initiate friendships with DHH students. Regarding assistive resources, participants noted that the use of visual tools—such as picture stories, drawing, writing, and alternative communication methods (e.g., pictures and drawings)—helps students express their social and emotional feelings more effectively and enhances their participation within the classroom environment. One participant stated: “*The use of visual aids, picture stories, drawing, and writing all helps DHH students express their social and emotional feelings.”* (P13).

Based on the frequency and analysis of responses, most participants ranked the factors influencing the social and emotional performance of DHH students in descending order as follows:

**Family** **←** **Peers** **←** **Teachers** **←** **Communication Methods** **←** **Educational Environment and Assistive Resources**

This ranking reflects participants' awareness of the integrated role of family, school, and social factors in supporting the social and emotional development of DHH students.

### 6.4 Results for forth research question

To address the fourth research question, thematic analysis (TA) was employed to analyze participants' responses. The findings revealed several strategies that teachers may employ to enhance the social and emotional performance of DHH students. These strategies were organized into seven subthemes, as presented below:

#### 6.4.1 Facilitating emotional expression and regulating emotions in difficult situations

Participants' responses showed that enabling DHH students to express their emotions requires the provision of safe and multiple channels of communication, including total communication, sign language, and visual means such as drawing, writing, and picture stories. Participants emphasized that giving students space to express themselves freely without judgment is a key factor in promoting psychological safety. As one participant stated: “*Total communication, writing, and drawing make students feel more comfortable expressing their emotions without fear”* (P4). Other participants highlighted the importance of a positive relationship with the teacher as a primary gateway to emotional expression. One participant noted: “*Trust and feeling comfortable with the teacher are the main reasons for expressing oneself freely*” (P15). Another participant stressed the importance of validating students' emotions, stating: “*It is essential that they feel their rights are recognized and that their emotions and thoughts are heard”* (P6). These findings suggest that emotional expression among DHH students is not linked to a single mode of communication, but rather to an integrated support system based on acceptance, visual communication, and emotional containment, particularly from teachers.

On the other hand, participants' responses indicated that emotional regulation among DHH students requires systematic and continuous training, rather than temporary intervention at the moment of emotional escalation. Participants emphasized the importance of teaching students to identify their emotions, clarifying behavioral expectations, and using strategies such as modeling and role-play. One participant explained: “*Students must be trained to recognize their emotions and understand behavioral expectations before the emotional situation occurs, not during it”* (P9). Some participants also stressed the teacher's role as an influential behavioral model in emotional regulation. As one participant stated: “*The teacher should be a role model, and the teacher's emotional regulation is reflected directly in the student”* (P11). These findings indicate that emotional expression and emotional regulation are acquired skills that require a supportive educational environment characterized by consistency, understanding, and positive guidance.

#### 6.4.2 Coping with frustration and academic failure

The analysis showed that helping DHH students cope with feelings of frustration or academic failure requires reframing failure as part of the learning process. Participants stressed the importance of breaking tasks into small, achievable steps and focusing on progress rather than final outcomes. One participant stated: “*We turn mistakes into opportunities for learning, and we focus on improvement rather than the final result”* (P11). Another participant supported this view by saying: “*They should be helped by setting alternative plans for success, presenting success stories after failure, providing additional lessons, and recognizing and praising their efforts, not only their results”* (P1). Some participants also highlighted the importance of individualized learning plans in addressing the academic frustrations these students may face. Other participants emphasized the role of psychological support, continuous encouragement, and allowing students to express emotions related to academic failure, all of which contribute to strengthening their resilience. These findings indicate that responding positively to academic failure can reduce frustration and enhance students' intrinsic motivation.

#### 6.4.3 Participation in programs and activities that support psychological adjustment

Most participants indicated that participation in school programs and activities plays an effective role in enhancing the psychological adjustment of DHH students, particularly individual and group counseling programs, extracurricular activities, and artistic and sports activities. One participant noted: “*Extracurricular activities and counseling programs give students an opportunity to express themselves and build self-confidence”* (P15). Other participants emphasized the importance of providing students with leadership roles and celebrating their achievements because of their positive effect on promoting competence and self-satisfaction. One participant stated: “*Programs that allow them to take leadership roles should be strengthened, such as participation in practical projects and classroom activities that require preparing displays and corners”* (P13). Another participant referred to assigning responsibilities inside and outside the classroom, saying: “*One teacher assigned the role of class monitor to two DHH students, and we noticed a positive difference in the two students' self-confidence and courage in speaking with others”* (P2).

#### 6.4.4 Using sign language and technological methods

Most participants reported that sign language and technological tools represent essential means for improving the interaction of DHH students with others. They emphasized that sign language is a rich emotional language, whereas applications and assistive devices serve as bridges for communication with hearing individuals. One participant explained: “*Sign language* + *technology* = *better communication and greater confidence, because sign language is a living language full of emotions through facial and body expressions, while technology is a bridge of communication with hearing people. This combination enables students to express themselves and interact effectively in all aspects of life. If both are integrated, interaction becomes broader and confidence in communication becomes higher among DHH students”* (P6). These findings indicate that combining sign language with assistive technologies contributes to expanding opportunities for social interaction and reducing feelings of isolation.

#### 6.4.5 Encouraging friendship formation and addressing bullying and social exclusion

Participants indicated that encouraging DHH students to form new friendships requires providing group activities, cooperative learning groups, and a school environment based on acceptance and respect. One participant expressed this as follows: “*Cooperative activities and extracurricular participation break barriers and help build friendships”* (P14). Participants also emphasized that intentionally planned social inclusion contributes to strengthening students' sense of belonging and positive integration into the school community. At the same time, the findings showed that addressing bullying experienced by students at school requires multiple strategies, including school-wide awareness, teaching safe self-advocacy and response skills, and providing psychological and counseling support. One participant stated: “*We need anti-bullying awareness programs and to teach students how to defend themselves confidently and safely, not through revenge”* (P1). Another participant highlighted the importance of establishing a team of supportive friends within the school: “*A team of supportive friends should be formed under the active supervision of the student counselor by promoting skills for building confidence and social competence, as well as offering individual and group sessions to strengthen students' psychological support.”* (P2). Another participant stressed the importance of avoiding pity, stating: “*When dealing with DHH students, it is essential not to show pity toward them, but rather to support them in taking responsibility just like their hearing peers.”* (P11). These findings confirm that addressing bullying should not be limited to the student alone, but rather requires an institutional and collaborative response involving teachers, school administration, and families.

#### 6.4.6 Developing cooperation and teamwork skills

Participants explained that developing cooperation skills among DHH students can be achieved through group projects, clear role distribution, and the use of supportive communication tools. One participant stated: “*Clearly dividing roles and assigning appropriate group projects helps the student participate effectively”* (P11). Another participant added: “*They should be distributed into mixed groups, rewarded for teamwork, and have their roles within the group clearly explained”* (P2). These findings suggest that teamwork contributes to strengthening social skills and building self-confidence.

#### 6.4.7 School initiatives and promoting community acceptance

Participants' responses showed that school initiatives, such as awareness workshops, teaching sign language to hearing students, and involving families, contribute to enhancing the acceptance of DHH students within the school community. One participant stated: “*Awareness workshops and teaching sign language made a clear difference in students' acceptance”* (P10). Another participant noted: “*Workshops were held at the beginning of this year to explain the developmental, linguistic, psychological, and social characteristics of hard-of-hearing students, and we observed a noticeable effect on general education teachers' acceptance and understanding of hard-of-hearing students”* (P5). A third participant added: “*It is essential to raise awareness in the school community and educate them about hearing disability and its characteristics. We also work on teaching the basics of sign language, in addition to community partnership by inviting successful DHH role models.”* (P13). These findings reflect the need to adopt sustainable institutional initiatives that promote a culture of acceptance and inclusion within schools.

Based on the foregoing, the qualitative analysis indicates that enhancing the social and emotional performance of DHH students depends on an integrated system that includes emotional support, effective communication, the roles of families and peers, school programs, and a supportive educational environment, all of which contribute to more effective social and psychological inclusion.

### 6.5 Results for fifth research question

To ensure a deeper level of integration between the quantitative and qualitative strands, the findings are interpreted using an explanatory approach in which qualitative evidence is used not only to contextualize but also to extend and reinterpret the quantitative results. The quantitative findings indicated that the overall level of social and emotional performance among DHH students was moderate (M = 3.64). While this result suggests a general mid-level of functioning, the qualitative findings provide a critical interpretive insight: this moderate level does not reflect uniform performance across domains, but rather a context-dependent pattern in which students perform relatively well in structured, supported situations, yet struggle in dynamic or unpredictable contexts.

This insight could not have been derived from either dataset independently. The quantitative data alone identifies the level as “moderate,” but does not explain the underlying pattern of variability. Conversely, the qualitative data describes contextual influences but does not quantify their overall impact. When integrated, the findings reveal that the “moderate” level is in fact the result of asymmetrical performance, where strengths in socially supported and emotionally scaffolded situations coexist with difficulties in spontaneous emotional regulation and adaptive responses for DHH students.

Specifically, higher mean scores in the quantitative scale items- such as teacher support, expression of joy, and communication through sign language or alternative means- are not merely indicators of generally strong performance. Rather, when interpreted alongside the qualitative findings, they reflect the effectiveness of structured support systems—including family encouragement, teacher and peer guidance, and accessible communication methods—in enabling DHH students to function successfully within predictable environments.

Participants consistently emphasized that family environments characterized by acceptance, encouragement, and emotional security enhance students' confidence and willingness to engage socially and express their emotions. When this insight is linked to the quantitative findings, it helps explain the relatively higher mean scores observed in items related to positive interaction, cooperation, and emotional expression. Similarly, the influence of peers—highlighted in the qualitative findings—provides a direct explanation for the higher performance observed in social interaction-related items, such as cooperation and friendship formation. Positive peer relationships foster a sense of belonging and social engagement, which is reflected in higher mean scores in these areas. This indicates that social performance improves when students are embedded in supportive interpersonal environments.

The teacher's role emerges as a central integrative factor linking both datasets. The highest quantitative mean score was associated with students' responsiveness to emotional support from teachers. Qualitative findings clarify that this is not merely a general tendency, but rather the result of strong teacher-student relationships characterized by trust, emotional support, and psychological safety. Teachers function as facilitators of communication and role models for emotional regulation, which explains why teacher-related items achieved the highest scores. This demonstrates that structured emotional support within the classroom directly enhances students' observable performance.

Communication methods also represent a key explanatory mechanism connecting both phases. Quantitatively, students showed relatively strong performance in items related to emotional expression through sign language or alternative means. Qualitatively, however, it became evident that communication effectiveness is context-dependent. While appropriate communication tools enable students to express emotions in structured or familiar situations, challenges persist in dynamic or unpredictable contexts. This explains the lower quantitative scores in items related to emotional flexibility and conflict management. Thus, communication is not only a facilitating factor but a foundational condition that determines the extent to which students can translate their emotional abilities into actual performance.

Furthermore, the educational environment and assistive resources provide an additional layer of explanation. Qualitative findings indicate that supportive and inclusive environments enhance students' sense of safety, confidence, and belonging. The availability of assistive resources—such as visual tools, alternative communication methods, and structured activities—also explains the relatively strong performance in emotional expression. However, the persistence of a moderate overall mean suggests that these supportive conditions are not consistently available across all contexts, resulting in variability in students' performance.

In contrast, lower mean scores in areas such as emotional regulation, conflict resolution, and emotional stability under pressure are not simply indicate an individual deficit among these students. Instead, the qualitative findings reveal that these challenges emerge primarily in situations where external supports are reduced or absent, such as spontaneous peer interactions, unfamiliar social contexts, or emotionally demanding situations. This suggests that students' difficulties are not inherent limitations, but are closely tied to contextual constraints, particularly communication barriers and variability in environmental support. Accordingly, while students are able to express their emotions effectively in familiar or structured contexts—especially when supported by appropriate communication methods—they tend to encounter difficulties in complex or unexpected situations that require rapid emotional adjustment. This, in turn, explains the discrepancy observed between relatively strong basic emotional expression and weaker performance in more advanced emotional regulation skills.

This integrated interpretation reframes the quantitative finding of a “moderate level” into a more nuanced understanding: DHH students demonstrate conditional competence, where performance is highly dependent on the availability of supportive structures. This concept extends the quantitative results by highlighting that improving social and emotional performance is less about addressing individual deficits alone and more about strengthening environmental supports and adaptive communication systems.

The qualitative findings further clarify that the aspects that appeared with lower scores in the quantitative assessment—such as emotional flexibility in unexpected situations, the ability to resolve simple conflicts, anger regulation, and emotional stability under pressure—should not be understood merely as individual weaknesses in the student. Rather, they should be interpreted in light of the surrounding contexts. These skills require continuous training, diverse social experiences, effective communication models, and stable psychological and educational support. If a student experiences difficulty rapidly understanding what is happening around them, expressing emotions immediately, or interpreting nonverbal social cues, then their responses in unexpected situations may become less flexible and more tense. Thus, the qualitative findings not only explain why social and emotional performance was at a moderate level, but also reveal the specific areas that require more focused and in-depth intervention.

It is also noteworthy that the strategies proposed by participants in response to Research Question 4 represent, in essence, a practical response to the contextual factors revealed in the qualitative findings, while simultaneously providing an additional explanation for the quantitative results. Calls for facilitating emotional expression, training students in emotional regulation, helping them cope with frustration and academic failure, encouraging participation in programs and activities, using sign language and technological tools, addressing bullying, building friendships, promoting cooperation, and strengthening community acceptance all indicate that social and emotional performance for DHH students is shaped by an integrated system of practices and everyday experiences it can be understood as targeted responses to this conditional pattern of performance. These strategies directly address the gap between supported and unsupported contexts, thereby offering a pathway to move students from context-dependent functioning toward more generalized social and emotional competence.

Accordingly, it can be argued that the relationship between the quantitative and qualitative findings in this study is integrative and explanatory. The quantitative findings revealed the level of social and emotional performance among DHH students, whereas the qualitative findings uncovered the contexts and factors that influence, elevate, or constrain this level. Thus, the findings confirm that the social and emotional performance of DHH students is not formed in isolation from context; rather, it is the product of an ongoing interaction between the individual characteristics of the deaf or hard-of-hearing student and the family, school, communicative, and social environments in which they live. This means that the moderate level shown in the quantitative findings does not represent a fixed or final state, but rather a level that can be developed and improved if these contextual factors are addressed systematically and intentionally. This interpretation provides a more comprehensive understanding than either dataset alone and aligns with the assumptions of a sequential explanatory mixed-methods design. It also underscores the importance of designing interventions that extend support beyond structured settings to enhance DHH students' ability to function effectively across diverse and dynamic social environments.

## 7 Discussion

The aim of this mixed-methods study was to investigate teachers' perceptions of the social and emotional performance of DHH students. The findings showed that teachers' perceptions—regardless of their experience, academic qualifications, the educational stage in which they worked, or the category of students they teach—indicated that the level of social and emotional performance among DHH students falls within the moderate range. The interviews further showed that all participating teachers were convinced that supporting students' social and emotional learning was not merely a secondary aspect of their work, but rather an essential component of their educational role.

This perception is linked to the impact of hearing loss on the social and psychological dimensions of DHH individuals. Although most of these individuals demonstrate levels of intelligence comparable to those of their hearing peers (Maller and Braden, 2011), numerous studies have shown that they face greater social and emotional challenges than hearing peers (Antia et al., 2011; Batten et al., 2014; Punch and Hyde, 2011; Rieffe, 2011). Research in cognitive, linguistic, social, and emotional domains also indicates that DHH individuals may encounter difficulties in accessing social learning in a predominantly hearing world, which may lead to delays in their social and emotional development (Marschark and Knoors, 2012; Michael et al., 2019). In this context, Bennett et al. (2022) identified a range of social and psychological difficulties experienced by DHH individuals. Similarly, Wiefferink et al. (2012) found that DHH children may experience greater problems in emotion regulation, lower social competence, and more difficulties interacting with age-matched hearing peers. Likewise, Brown and Cornes (2015), in their study of DHH adolescents, reported higher rates of internalizing problems such as social isolation, anxiety, and depression, in addition to externalizing problems such as aggression and rule-breaking.

In light of these challenges, the findings of the present study are consistent with previous studies emphasizing the importance of adopting individualized approaches by teachers and specialists when assessing and addressing the social and emotional needs of DHH students. Foster and Cue (2009) described teachers' important role in supporting students' social and emotional development through the promotion of self-advocacy skills and coping abilities. Likewise, Norman and Jamieson (2015) found that many teachers of DHH students view themselves as the only professionals in the educational system who possess both the specialized knowledge and the long-term relationship with students necessary to support them in facing social and emotional challenges. The nature of this relationship fosters trust and openness between teacher and student, thereby enhancing the teacher's ability to provide social and emotional support.

Accordingly, there appears to be substantial value in social and emotional learning programs for DHH students. Empirical evidence suggests that participation in SEL interventions is associated with significant improvements in students' self-perceptions, interpersonal attitudes, positive social behaviors, and overall engagement with school compared with peers who do not receive such interventions (Durlak et al., 2011). Furthermore, research has consistently demonstrated the sustained effectiveness of SEL programs across diverse educational contexts, including general education classrooms (Kelly et al., 2004), special education settings (Kam et al., 2004), and learning environments serving DHH students (Greenberg and Kusché, 1998). The findings of the present study support this perspective, as participating teachers acknowledged the necessity of supporting students' social and emotional learning, and both the scale and interview findings showed that they already provide direct support in this domain. However, despite this clear awareness of the importance of social and emotional learning, some participants in the present study reported lacking the training needed to provide such support effectively. Teachers indicated in the interviews that they often rely on personal experience and professional intuition when dealing with students' social and emotional issues. They also expressed a need for practical skills, applied training, and programs specifically designed for the nature of their work. This finding is consistent with teacher preparation programs for teachers of DHH students, which typically focus on language, curriculum, and auditory-verbal skill development, while largely lacking mandatory content related to teaching social and emotional learning (Canadian Association of Educators of the Deaf Hard of Hearing, 2009). This, in turn, points to the need to reconsider teacher preparation programs so that they include systematic training on strategies for supporting social and emotional learning (Norman and Jamieson, 2015).

In this context, previous studies suggest that teachers' beliefs play an important role in delivering social and emotional learning at school. Brackett et al. (2012) showed that teachers' beliefs regarding their competence in teaching social and emotional learning influence their level of comfort in implementing it. Teachers' commitment to ongoing professional development in this area also enhances their ability to apply effective strategies to support this type of learning. In addition, teachers' perceptions of the extent to which school culture supports social and emotional learning programs may affect the quality of implementation. Accordingly, teachers' beliefs about their professional competence, commitment to professional development, and support from the school environment constitute important predictive indicators of the implementation of social and emotional learning for DHH students.

The present study also showed that the educational environment represents an important factor influencing the social and emotional performance of DHH students. This is consistent with Norman and Jamieson (2015), who found that school cultures may either support or hinder the teaching of social and emotional learning. In that study, only about 45% of teachers reported receiving the support needed when delivering social and emotional learning in the schools where they worked. This variation may be attributable to differences in school environments, especially given that many teachers work across multiple schools and are therefore exposed to differing school cultures. Teachers in that study also emphasized the importance of supportive school leadership in fostering an inclusive and supportive environment for social and emotional learning. In a similar vein, Garberoglio et al. (2012) found that teachers' perceptions of the collective efficacy of the school environment—that is, the culture—were related to their scores on measures of self-efficacy, suggesting a close relationship between school culture and the professional competence of teachers of DHH students, which in turn is reflected in their implementation of social and emotional learning skills.

On the other hand, the type of educational setting—inclusive education vs. special programs—may affect the social interactions and the social and emotional performance of deaf or hard-of-hearing students (Lukomski, 2007). Stinson et al. (1996) found that deaf students in both mainstream and special programs reported feeling greater emotional security when interacting with peers who also had hearing loss than when interacting with hearing peers. In contrast, Van Eldik (2005) found that deaf adolescents in special programs may exhibit higher levels of withdrawn behavior, somatic complaints, and anxiety or depressive feelings than their peers in mainstream education. Kluwin et al. (2002), however, concluded that there is insufficient conclusive evidence to support definitive claims about social and emotional outcomes based solely on school type. This is consistent with the present findings, which suggest the importance of having deaf or hard-of-hearing peers within the school who share similar personal challenges and can form supportive groups to counter bullying and social exclusion.

From a broader perspective, the type of school environment may play a dual role for DHH students. Schools may represent supportive contexts that promote mental health, or they may increase psychological risks as a result of experiences such as peer bullying (Bouldin et al., 2021; Runions et al., 2020). Although the importance of inclusive education for students with disabilities has been internationally recognized (Kishida et al., 2022), simply placing DHH students in mainstream settings does not necessarily guarantee positive educational or social outcomes, because positive interaction and peer acceptance do not occur automatically (Xie et al., 2014). Rather, they require active participation from students, their peers, and the broader school community.

Common challenges in the inclusion of DHH students include limited awareness among hearing peers and teachers about the nature of hearing loss, as well as limited teacher skills in managing hearing devices or supporting communication within the classroom (Berndsen and Luckner, 2012). Limited access to auditory information in classroom settings may also hinder academic learning and social interaction (Edmondson and Howe, 2019). As a result, most general education teachers recognize that they are integral to facilitating students' mental health and wellbeing, yet they lack the foundational training required to provide this support (Kidger et al., 2010). These findings are supported by an Australian study that examined mainstream teachers' perceptions of their role as facilitators of the social and emotional development of DHH students, which pointed to the need for classroom resources that equip teachers with strategies and knowledge to support these students' wellbeing (Furness et al., 2019). Therefore, schools need to take into account the unique needs and experiences of DHH students to ensure positive outcomes in education and psychological and social wellbeing (Kishida et al., 2022).

The present findings also indicate that problems associated with deafness or hearing loss, such as social isolation and exclusion, are among the major challenges that may hinder emotional expression, emotional regulation, friendship formation, and social inclusion. This is consistent with Norman and Jamieson (2015), who reported that social isolation was one of the most salient concerns identified by teachers of DHH students, given its broad impact on academic achievement, social interaction, and even family relationships. Thus, feelings of isolation and loneliness among DHH students in inclusive settings may adversely affect their social and emotional performance.

The qualitative findings further showed that the family is one of the most important factors influencing the social and emotional performance of DHH students. This is consistent with several studies emphasizing that early parent-child interactions play a critical role in children's linguistic, social, and emotional development (Devine and Hughes, 2018; Hintermair et al., 2017; Tamis-LeMonda et al., 2019). It is worth noting that most DHH children have hearing parents who may not have fully accessible means of communication with them, which may affect their social and emotional development (Kawar et al., 2022). Guralnick (2011) proposed a theoretical framework highlighting the role of early parent–child relationships in supporting developmental progress, arguing that developmental support occurs through the everyday activities of family life. The success of early intervention depends on parents' ability to adapt to their child's unique developmental and behavioral characteristics and to respond to the child's needs through affection, responsiveness to interests, the establishment of shared attention, and adjustment of language to the child's receptive language level. In this regard, Smit et al. (2023) pointed out that the role of social support sources changes across developmental stages: parents serve as the primary guides in childhood, whereas peers become increasingly important during adolescence.

It is also important to recognize the heterogeneity within the DHH population, as several factors may affect their social and emotional performance, including the severity, cause, and stability of hearing loss, rehabilitation procedures, the use of hearing devices, and different communication modes (Van Beijsterveldt and van Hell, 2009). Studies have shown that higher language abilities, whether in spoken language or sign language, are associated with lower levels of social and emotional difficulties (Kawara and Michaela, 2025). Hall et al. (2017) also found that DHH children who use sign language may demonstrate typical social and emotional performance compared with their peers.

With respect to differences associated with the study variables, the findings differ from those of Norman and Jamieson (2015)), who found that less experienced teachers tended to show greater commitment to professional development in social and emotional learning, whereas more experienced teachers tended to demonstrate higher self-efficacy and greater comfort in teaching this area, which may reduce their inclination to pursue further professional development. Interest in professional development among teachers at the beginning of their careers may reflect their desire to improve their teaching practices and support student wellbeing. That study also indicated that professional colleagues represent an important source of support for teachers of DHH students when dealing with students' social and emotional challenges, and in the absence of such support teachers may experience a form of professional isolation. This discrepancy may be explained by several contextual factors related to the educational environment in which the present study was conducted. Unlike the context examined by Norman and Jamieson (2015), teachers in the current study appear to operate within relatively similar institutional conditions, including comparable training opportunities, professional expectations, and shared practices for supporting DHH students. Such contextual consistency may contribute to a convergence in teachers' perceptions regardless of their educational qualifications, years of experience, the educational stage in which they teach, or the category of students they teach. In addition, the nature of working with DHH students often requires teachers to adopt collaborative practices and rely on shared pedagogical strategies, which may reduce differences in perceptions across professional backgrounds. As a result, teachers may develop relatively similar understandings of students' social and emotional needs through daily classroom interactions and collective professional experiences. Therefore, the absence of statistically significant differences across the study variables may reflect the influence of a common professional culture and shared educational practices within inclusive educational settings.

Overall, there is broad agreement that DHH students should leave the educational system with not only academic knowledge, but also social and emotional skills that enable them to interact positively with others, make responsible decisions, and engage in healthy behaviors (Association for Supervision Curriculum Development, 2007). Accordingly, promoting social and emotional learning among DHH individuals requires greater attention from both practitioners and researchers to ensure that these students leave school equipped with the knowledge and skills necessary to live, learn, and integrate into a changing and predominantly hearing global society (Luckner and Movahedazarhouligh, 2018).

### 7.1 Conclusion and recommendations

The present study concluded that the social and emotional performance of DHH students in inclusive schools was at a moderate level from teachers' perspectives. This indicates that these students possess a number of social and emotional skills, while still requiring further support and reinforcement in some areas related to emotional expression, psychological flexibility, and social interaction. The qualitative findings showed that social and emotional performance is influenced by a set of interrelated contextual factors, most notably the roles of family, peers, and teachers, as well as communication methods, the educational environment, and assistive resources. The findings also indicated that enhancing this performance requires an integrated system of educational and social practices, including enabling students to express their emotions, supporting emotional regulation, encouraging social inclusion, and providing a school environment based on acceptance and effective communication. Accordingly, the study highlights the importance of improving teacher preparation and professional development programs, activating family-school partnerships, and adopting inclusive practices that respond to the social and emotional needs of DHH students in ways that contribute to their psychological and social wellbeing and academic success. Potential directions for future research include adopting a multi-source approach involving students, general education teachers, and teaching assistants, and expanding the scope of research to examine how social and emotional learning is reflected in educational practices across specific areas of special education.

### 7.2 Study limitations

Despite the importance of the findings of this study in understanding teachers' perceptions of the social and emotional performance of DHH students, several limitations should be considered when interpreting the results. One limitation of this study relates to the psychometric validation procedure for the scale. The exploratory factor analysis (EFA) and related reliability analyses were conducted using the same sample (*N* = 102) employed for the primary quantitative analyses. While this approach was necessary to ensure an adequate sample size for stable factor extraction, it may introduce a potential risk of sample-specific bias and a form of circular validation, whereby the factor structure is derived and evaluated using the same dataset. This, in turn, limits the ability to establish independent validation of the instrument. Accordingly, the findings should be interpreted with caution. Future research is recommended to replicate the factor structure using an independent sample and to further validate the instrument through confirmatory factor analysis (CFA).

In addition, several contextual and methodological limitations should be considered when interpreting the findings. First, the study relied on a small, context-specific qualitative sample that was purposively selected to generate in-depth understanding rather than statistical representation. Accordingly, the findings are not intended to be generalized probabilistically, but rather to provide analytical and contextual insights into how teachers perceive social and emotional performance within a specific social, cultural, and educational context. Second, the study was limited by the nature of the sample, as it focused exclusively on female teachers of DHH students in inclusive schools. This may limit the generalizability of the findings to all teachers or to other educational contexts, such as specialized schools for deaf students or educational settings in other regions. In addition, the sample size and its concentration on a specific group of female teachers may not necessarily reflect all perspectives or professional experiences related to supporting the social and emotional performance of this group of students. Third, the study employed a mixed-methods design that combined quantitative and qualitative data, with a strong emphasis on the qualitative component. Although this design provided a more comprehensive understanding of the topic, reliance on teachers' self-reported qualitative responses may have been influenced by perceptual or social desirability biases. Similarly, the qualitative findings derived from interviews reflect participants' experiences and views within their own professional contexts, which may limit their transferability to all educational environments. Finally, reliance on self-reported narratives may also have shaped the findings through cultural norms surrounding emotional expression and participants' personal comfort with disclosure. Rather than viewing this solely as a methodological weakness, it should be acknowledged as an inherent feature of qualitative research, which prioritizes participants' subjective meanings as a legitimate source of knowledge. Accordingly, the findings should be understood as theoretically valuable and contextually grounded, rather than as universal psychological patterns. Future research may build on this work by broadening institutional contexts, incorporating longitudinal perspectives, and exploring comparative cases to examine how understandings of and adaptation to social and emotional performance among DHH students evolve across different educational and cultural settings.

## Data Availability

The original contributions presented in the study are included in the article/Supplementary material, further inquiries can be directed to the corresponding author/s.
