# Supplementary material for: Social and emotional performance of deaf and hard-of-hearing students in inclusive schools: a mixed-methods analysis of teachers' experiences in Saudi Arabia
Source: Front Psychol. 2026 May 29;17:1828336. doi: 10.3389/fpsyg.2026.1828336 (PMC13260558; doi:10.3389/fpsyg.2026.1828336)
Supplement: Supplementary file 1 [file Supplementary_file_1.docx]

**Appendix A**

**Dear Teacher,**

Peace and blessings be upon you.

The researcher is conducting a study on the social and emotional performance of deaf and hard-of-hearing students from teachers’ perspectives. This study is intended for scientific research purposes and to contribute to the improvement of educational practices in the field of deaf and hard-of-hearing education.

You are kindly requested to read the following statements carefully and select the response that best reflects your opinion. Please note that your responses will be treated with full confidentiality and will be used solely for scientific research purposes.

Thank you very much for your valued cooperation.

**Part I: Background Information**

**Educational Qualification:**

- Bachelor’s degree in Special Education
- Bachelor’s degree in General Education
- Master’s degree
- Other (....................)

**Years of Experience in Teaching Deaf and Hard-of-Hearing Students:**

- Less than 1 year
- 1–5 years
- More than 5 years

**Educational Stage Currently Taught:**

- Early Childhood (Grades 1–3)
- Primary Stage (Grades 4–6)
- Intermediate Stage
- Secondary Stage

**Teaching Category:**

- Deaf students
- Hard-of-hearing students
- Deaf and hard-of-hearing students

**Second: Scale Statements**

| **No.** | **Item** | **Always** | **Often** | **Sometimes** | **Rarely** | **Never** |
| --- | --- | --- | --- | --- | --- | --- |
| **Axis One: Social Performance** | | | | | | |
| 1 | The student interacts positively with classmates in the classroom. |  |  |  |  |  |
| 2 | The student participates in group activities at school. |  |  |  |  |  |
| 3 | The student is able to form friendships with peers. |  |  |  |  |  |
| 4 | The student demonstrates the ability to cooperate with others. |  |  |  |  |  |
| 5 | The student expresses feelings in front of others. |  |  |  |  |  |
| 6 | The student shows initiative in situations requiring direct communication. |  |  |  |  |  |
| 7 | The student takes the initiative to help classmates when needed. |  |  |  |  |  |
| 8 | The student maintains positive relationships with friends. |  |  |  |  |  |
| 9 | The student shows the ability to resolve simple conflicts with classmates. |  |  |  |  |  |
| 10 | The student is socially accepted among peers. |  |  |  |  |  |
| 11 | The student demonstrates socially responsible behavior. |  |  |  |  |  |
| 12 | The student possesses positive social interaction skills. |  |  |  |  |  |
| 13 | The student responds positively to emotional support from the teacher. |  |  |  |  |  |
| 14 | The student is able to express emotions using sign language or alternative means. |  |  |  |  |  |
| 15 | The student can easily express feelings of joy when succeeding in academic tasks. |  |  |  |  |  |
| **Second axis: Emotional performance** | | | | | | |
| 16 | The student avoids getting into problems with classmates. |  |  |  |  |  |
| 17 | The student demonstrates the ability to regulate emotions in different situations. |  |  |  |  |  |
| 18 | The student demonstrates emotional stability when facing academic pressure. |  |  |  |  |  |
| 19 | The student deals calmly with difficult situations. |  |  |  |  |  |
| 20 | The student expresses feelings of joy and happiness naturally. |  |  |  |  |  |
| 21 | The student shows anxiety or tension in new situations. |  |  |  |  |  |
| 22 | The student is able to overcome feelings of frustration after failure. |  |  |  |  |  |

**Thank you**
