# Supplementary material for: Social and emotional performance of deaf and hard-of-hearing students in inclusive schools: a mixed-methods analysis of teachers' experiences in Saudi Arabia
Source: Front Psychol. 2026 May 29;17:1828336. doi: 10.3389/fpsyg.2026.1828336 (PMC13260558; doi:10.3389/fpsyg.2026.1828336)
Supplement: Supplementary file 2 [file Supplementary_file_2.docx]

**Appendix B**

**Individual Interview Guide**

Thank you for agreeing to participate in this interview. The purpose of this discussion is to gain a deeper understanding of teachers’ perceptions of the social and emotional functioning of deaf and hard-of-hearing students, as well as the strategies that may help enhance it. There are no right or wrong answers; what matters most is your honest perspective. You are free to skip any question you prefer not to answer, and all responses will remain completely confidential.

1. From your perspective, what are the most effective ways that help deaf and hard-of-hearing students express their emotions appropriately?
2. How can teachers help deaf and hard-of-hearing students regulate their emotions in challenging situations?
3. What role do family members and peers play in enhancing the self-confidence of deaf and hard-of-hearing students and increasing their self-satisfaction?
4. How can students be supported in coping with feelings of frustration or academic failure?
5. What programs or activities do you believe can enhance the psychological adjustment of deaf and hard-of-hearing students within the school environment?
6. How do students benefit from sign language or assistive technologies (such as applications or assistive devices) in improving their interactions with others?
7. What approaches can encourage deaf and hard-of-hearing students to form new friendships?
8. What strategies can help students deal with situations of bullying or social exclusion that they may experience?
9. How can collaboration and teamwork skills be enhanced among deaf and hard-of-hearing students?
10. What initiatives can contribute to increasing the acceptance of deaf and hard-of-hearing students within the school community?
11. Can you describe a situation in which a teacher used a strategy to improve the social and emotional performance of her students?
12. From your perspective as a teacher, which strategies are most effective in helping these students improve their social and emotional performance? (Please rank the following strategies in order of importance by placing a number next to each one.)

( ) Family
( ) Teachers
( ) Peers
( ) Technological tools
( ) Academic achievements
( ) Ignoring negative behaviors

Thank you
